# Supplementary material for: Practical high-dimensional quantum key distribution protocol over deployed multicore fiber
Source: Nat Commun. 2024 Feb 23;15:1651. doi: 10.1038/s41467-024-45876-x (PMC10891113; doi:10.1038/s41467-024-45876-x)
Supplement: Supplementary file 1 — Supplementary Information [file 41467_2024_45876_MOESM1_ESM.pdf]

Practical High-Dimensional Quantum Key Distribution Protocol over deployed Multicore fiber

Mujtaba Zahidy, Ronny Müller, Leif K. Oxenløwe, and Michael Galili
Technical University of Denmark, Department of Electrical and Photonics Engineering, Ørsteds Pl., Kgs. Lyngby, Denmark, 2800

Domenico Ribezzo
Department of Physical and Chemical Sciences, University of L'Aquila, L'Aquila, Italy
Istituto Nazionale di Ottica, Consiglio Nazionale delle Ricerche (CNR-INO), Firenze, Italy, 50125 and
University of Naples Federico II, Napoli, Italy

Claudia De Lazzari, Ilaria Vagniluca, Nicola Biagi, and Tommaso Occhipinti
QTI S.r.l., Firenze, Italy, 50125

Tetsuya Hayashi
Optical Communications Laboratory, Sumitomo Electric Industries, Ltd., Yokohama, Japan, 244-8588

Dajana Cassioli
Department of Information Engineering, Computer Science and Mathematics, University of L'Aquila, L'Aquila, Italy and
National Laboratory of Advanced Optical Fibers for Photonics (FIBERS), CNIT, L'Aquila, Italy

Antonio Mecozzi and Cristian Antonelli
Department of Physical and Chemical Sciences, University of L'Aquila, L'Aquila, Italy and
National Laboratory of Advanced Optical Fibers for Photonics (FIBERS), CNIT, L'Aquila, Italy

Alessandro Zavatta
Istituto Nazionale di Ottica, Consiglio Nazionale delle Ricerche (CNR-INO), Firenze, Italy, 50125 and
QTI S.r.l., Firenze, Italy, 50125

Davide Bacco*
QTI S.r.l., Firenze, Italy, 50125 and
Department of Physics and Astronomy, University of Florence, Via Nello Carrara, Firenze, Italy, 50019

I. SUPPLEMENTARY NOTE 1: COMPARISON OF HI-D IMPLEMENTATION WITH 2-D COUNTERPART

In order to assess the gain of our 4D protocol, a 2D path-encoding scheme is implemented. The mutually unbiased bases of the $2D$ realization are simply defined as $\mathcal{X} = \{|A\rangle, |B\rangle\}$ and $\mathcal{Z} = \{(|A\rangle \pm |B\rangle)/\sqrt{2}\}$ where $|A\rangle$ and $|B\rangle$ are the two cores of the multi-core fiber (MCF). The $\mathcal{X}$ basis measurement scheme simplifies to a DWDM filter (for out-of-band noise suppression) and a detector per fiber core, whereas in the case of the $\mathcal{Z}$-basis, the measurement scheme does not change and is similar to the 4D case. We compare our results with this QKD system based on 2-dimensional path-encoding, showing an improvement of $\approx 118\%$ in terms of key generation rate at 22 dB of channel loss. At this channel loss, the estimated secret key rates for the 2D and 4D protocols are of 23.6 kbps and 51.5 kbps, respectively, corresponding to a gain in secret key rate by 118% in the 4D protocol, see Supplementary Fig. 1. Supplementary Table 1 summarizes the parameters and QBERs of the two implementations. Note that the measured gain is achieved in spite of the fact that the QBER value is smaller for all bases in the 2D protocol.

Parameter	2D	4D
μ_1 (μ_2)	0.36 (0.16)	0.36 (0.16)
p_{μ_1} (p_{μ_2})	0.81 (0.11)	0.81 (0.11)
$\text{QBER}_{Z_{\mu_1}}$	0.51%	2.15%
$\text{QBER}_{Z_{\mu_2}}$	0.86%	2.43%
$\text{QBER}_{X_{\mu_1}}$	2.15%	3.54%
$\text{QBER}_{X_{\mu_2}}$	2.43%	3.88%
p_{gate}	0.48	0.53

Supplementary Table 1: **Experimental parameters.** Measured QBERs, and achieved key rates in both bases, for the two transmitted bases in 2D and 4D. The measured channel loss is 22 dB.

* davide.bacco@unifi.it

The secret key length is estimated in a finite-block-size regime with security parameter ϵ_{sec}, correction parameter ϵ_{corr}, and block size n in 2D with[1]

$$\ell_{2D} \leq D_0^Z + D_1^Z[1 - h(\phi_Z)] - \lambda_{EC} - 6\log_2(21/\epsilon_{\text{sec}}) - \log_2(2/\epsilon_{\text{corr}}), \tag{1}$$

in 4D with[2]

$$\ell_{4D} \leq 2D_0^Z + D_1^Z[2 - H(\phi_Z)] - \lambda_{EC} - 6\log_2(21/\epsilon_{\text{sec}}) - \log_2(2/\epsilon_{\text{corr}}), \tag{2}$$

where D_0^Z and D_1^Z are the lower bounds of vacuum and single-photon events in the Z basis, $h(x) := -x\log_2(x) - (1-x)\log_2(1-x)$ is the Shannon entropy and $H(x) := -x\log_2(x/3) - (1-x)\log_2(1-x)$ is the Shannon entropy for $4D$ variables, ϕ_Z is the phase error rate upper bound, and λ_{EC} is the number of bits that are publicly disclosed during error correction[2]. $\epsilon_{sec} = \epsilon_{corr} = 10^{-12}$ have been set. We chose a block size of $n_Z = 10^8$ for both the 2D and 4D protocol, and we estimated for the 4D states an error reconciliation efficiency $f_{err,4D} = \lambda_{EC}/(n_Z * H_{4D}(\phi_Z)) = 1.06$, which is in line with the most recent results reported in the literature for the measured QBER values adopting the original cascade error correction protocol[3]. In the 2D protocol, $f_{err,2D} = \lambda_{EC}/(n_Z * H_{2D}(\phi_Z)) = 1.08$ has been adopted[4].

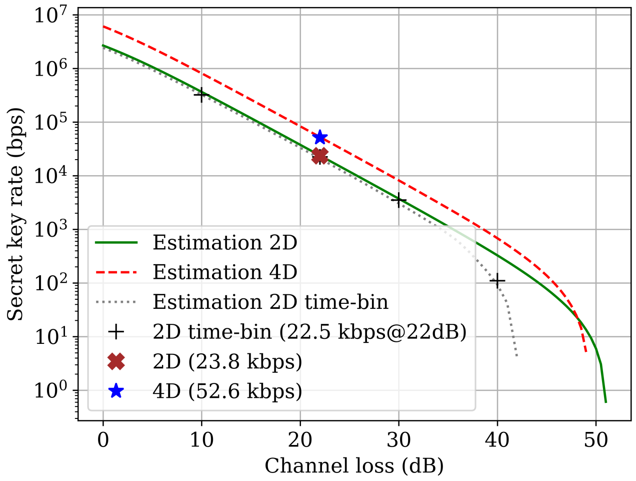

Supplementary Figure 1: **Experimental results.** Comparison of finite size secret key rate achievable with 2D time-bin, 2D path-encoding, and 4D hybrid systems with our data point at channel loss equivalent to 22 dB.

II. SUPPLEMENTARY NOTE 2: COMPARISON OF HI-D IMPLEMENTATIONS

Comparison of several High-dimensional quantum key distribution experiments. In Supplementary Tab. 2, relevant parameters of a QKD system, as reported in the references, are listed. The name of the protocol is reported as described in the reference.

Ref	ν[Hz]	D	Protocol	DoF	channel length [km]	η_{Ch} [dB]	η_{Rx} [dB]	η_{SPD} [dB]	QBER %	SKR [Kbps]
This work	483 M	4	Eff. BB84	Path-time	52	22	4	0.809	3.5	113
[5]	1 K	4	BB84	Path	0.3		24.5	12.2	10.25 ± 0.6	(4.31 ± 1.19) * 10^{-9}
[6]*	30	16	BB84	OAM	0.0005				13.4 ± 4<br>15.6 ± 7	7.5 * 10^{-6}<br>3.1 * 10^{-6}
[7]	4 K	14	BB84	OAM	0.002		35.1	1.87	10.5	6.8 * 10^{-3}
[2]	297.6 M	4	BB84	TB	25<br>65<br>105<br>145	5.1<br>14<br>23<br>31.5	2.5	9.2	3.4<br>3.4<br>4.9<br>7.9	37<br>24<br>5.5<br>0.42
[8]		3	DO-QKD	Energy-time	242				7.9	6 * 10^{-5}
[9]	1 M	1024	photon-efficient HDQKD	Energy-time	20			0.458	39.6	2700
[10]	312.5 M<br>625 M<br>1.25 G	16<br>8<br>4	DO-QKD	Energy-time	–<br>41<br>43	0.1<br>7.6<br>12.7	6	1.67	6.5<br>4.8<br>4.9	23000<br>5300<br>1200
[11]†	2.5 G	4	HD-QKD	TB	4<br>8<br>10<br>14<br>16.6	20<br>40<br>50<br>70<br>83		1.55	4.5	26200<br>11900<br>7710<br>3400<br>1070
[12]	60	4	HD-QKD	Path-OAM						
[13]				Energy-time					5	
[14]	80 M	4<br>5	HD-QKD	OAM	< 1		10		8.8<br>14	1.139<br>0.8606
[15]	5 K	4	BB84	Path		10<br>20	8		13	–<br>–
[16]	600 M	4	HD-QKD	OAM	1.2	1.2	10		14-18	37.85

Supplementary Table 2: **Comparison table.** Comparison of recent studies on HD QKD in terms of source repetition rate, dimension, implemented protocol, channel loss, and the final SKR. All the η values are presented as loss factors in the system and should be regarded as negative.
* The final SKR is not reported. This number corresponds to the sifted key rate.
† The SKR drops rapidly to zero after 18 dB.

III. SUPPLEMENTARY NOTE 3: NORMALIZED SECRET KEY RATE FOR SELECTED HIGH-PERFORMANCE IMPLEMENTATIONS

Comparison of the normalized secret key rate taking into account the source rate and the number of detectors used for data acquiring for a group of recent implementations, see Supplementary Tab. 3. Multi-pixel SNSPDs or multiplexing to four detectors is a practice employed to increase the rate. Supplementary Fig. 2 represents normalized SKR vs. channel loss.

IV. SUPPLEMENTARY NOTE 4: EXPERIMENTAL SETUP

In order to generate a secure key between remote users, it is necessary to follow a precise and well-defined protocol. First of all, two channels that are distinctly different in nature, the quantum channel and the classical channel, are required to implement the entire QKD protocol. In particular, while the classical channel is only required to convey classical information (and therefore can be chosen among many possible options, like fiber, free space, copper, etc..), the quantum channel has to transfer quantum states, of which it must preserve the main properties.

This limits considerably the selection of physical channels that are suitable for quantum communication. Optical fiber is one option, as it allows transmitting quantum states of light generated at optical frequencies. In the case of discrete-variable encoding technique (i.e., the degrees of freedom used to encode the quantum states are discrete), two mutually unbiased bases have to be created and randomly selected by the source (Alice). Subsequently, the receiver will randomly select one of the two bases to measure the quantum states, once these have been sent over the quantum channel. A sifting procedure followed by an information reconciliation protocol is then executed in the classical channel in order to select the final secret key. Our Hi-D quantum states belong to a four-dimensional extended Hilbert space resulting from the tensor product of a $2D$ time-bin and a $2D$ path-encoding space. The eight generated qudits, each encoding two bits of quantum information, form

Ref.	ν (Hz)	Protocol	channel (dB)	N_{Det_Z}	SKR (bps)	SKR/ν/N_{Det}
17	2.5 G	efficient BB84	2.5<br>19.6<br>55.1	16	115.8 M<br>2.6 M<br>233	0.002895<br>6.5e-5<br>5.825e-9
18	2.5 G	3 states BB84	1.58<br>16.34	14	64 Mbps<br>3Mbps	0.00183<br>8.57e-5
11	2.5 G	HD QKD (4D)	4<br>10<br>16.6	4	26.2<br>7.71<br>1.07	0.00262<br>0.000771<br>0.000107
This work	487 M	BB84<br>HD QKD	22	2	23.6<br>51.5	2.422e-5<br>5.286e-5

Supplementary Table 3: **Comparison with selected work.** Here we compare our SKRs with a group of selected works, keeping into account the state generation rate in the source and the number of detectors used in the key generation basis. We do not consider the detector efficiencies since they were not always reported. All the high-rate works employed high-efficiency superconductive nanowire single-photon detectors.

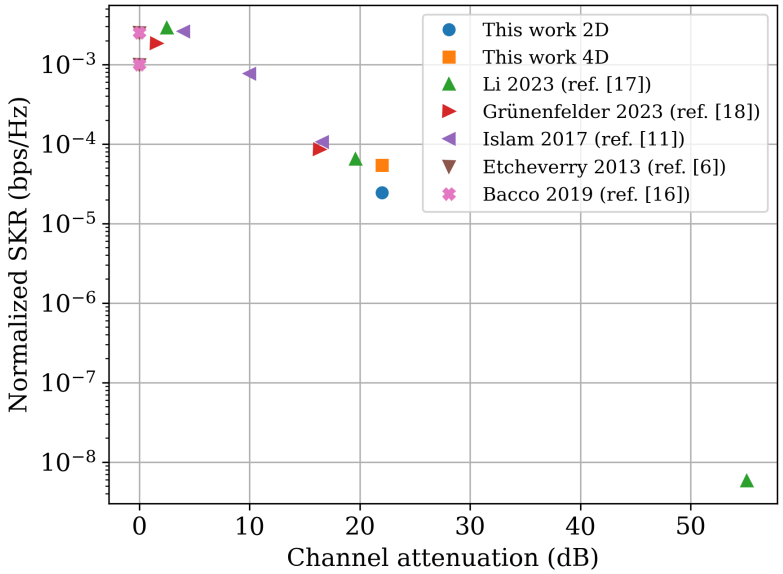

Supplementary Figure 2: **Comparison with selected work.** The secure key rates in the y-axis are normalized per the source generation rate and the number of detectors in the key generation basis. The x-axis expresses the attenuation of the utilized channel.

two orthogonal bases, as shown in Supplementary Fig. 3. In the $\mathcal{Z}$ basis, two synchronous pulses are transmitted in two fiber cores, so that one information bit is encoded in the time bin occupied by the two pulses, and another in the relative phase between them. In the $\mathcal{X}$ basis, two sequential pulses are transmitted in one fiber core only (while no pulse is transmitted in the other core), so that one information bit is encoded in the spatial path, and another in the relative phase between the two pulses (see Methods for implementation details). To retrieve the encoded information, we interfere the quantum pulses after traveling in the two cores. The main source of error and loss of visibility is the relative phase fluctuation of the two paths. Multicore fibers have unique features such as low relative phase[19] and polarization[20] drift and high core-to-core isolation[21] that make them highly attractive for classical and quantum applications. By confining all the cores in one cladding, signals are less susceptible to error, to the degree that a phase-locked loop (PLL) can eliminate the slow phase fluctuations and yield a high visibility interference result.

To compare the performance of our protocol in terms of secret key generation rate and error tolerance, we implemented a $2D$ path-encoding protocol which was also tested during the field trial. The two- and four-dimensional realizations were implemented using the experimental setup shown in Supplementary Fig. 4. This provides a fair comparison between $2D$ and $4D$ protocols. Note that according to the state formation presented above, a single 2D time-bin implementation would have an equal qubit generation rate as the hybrid time-path encoding. However, the 2D path-encoding can benefit from a rate twice as the 4D implementation, as each bin serves one qubit. Transmitter (Alice) prepares the qudit states starting from a $2D$ time-bin encoding source and expanding it in two paths, here, two cores of a multicore fiber. To compensate and damp

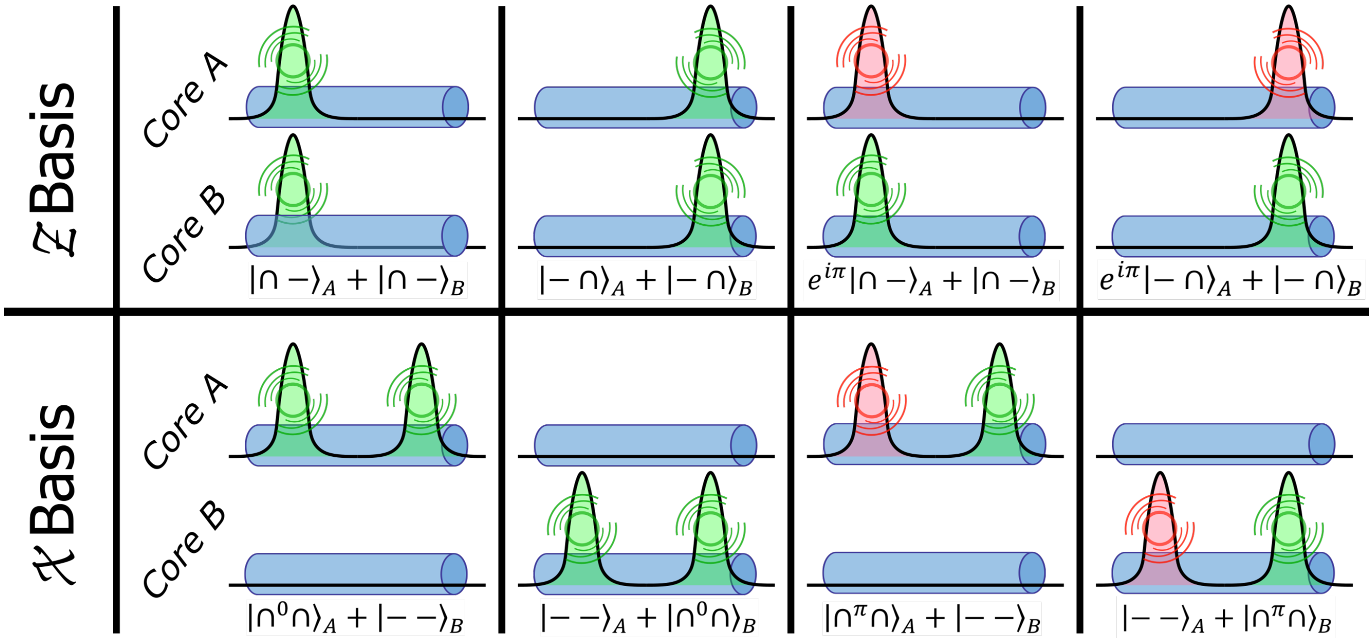

Supplementary Figure 3: **State Generation.** $\mathcal{Z}$ states are generated by blocking one bin of time-bin states in each core, while $\mathcal{X}$ states are generated by blocking one core for a complete time-bin state duration. In both cases, the application of the proper phase completes the state preparation. Here, the symbol "$\cap$" denotes the presence of a pulse in a time bin, while the absence of a pulse is denoted with the symbol "$-$". The subscript $X \in \{A, B\}$ denotes the core in which the time-bin state is injected. The superscript $\phi \in \{0, \pi\}$ is used to denote a ϕ-phase shift between sequential pulses in the same time-bin state. The color red is used for a π-phase difference.

phase fluctuations in the fiber, we adopt a dual-band phase control technique in which a second classical continuous wave (CW) *monitor* laser is multiplexed and co-propagated in fiber[19,22]. The two wavelengths, monitor and quantum, are selected to be 400 GHz apart to experience similar propagation effects along the fiber channel. A sensitive avalanche photodiode (APD) with 9 A/W responsivity was used to measure the variation of power after interfering the classical CW light traveling in the two cores and pass it as feedback to a phase-locked loop to preserve the visibility. This technique allows for high power contrast while keeping the Raman effect at a low level. Furthermore, the stabilization is completely classical without imposing any overhead to the quantum communication such as using a header or part of the qubits for drift/clock compensations[23], and hence does not affect the rate of the 4D system other than extra loss due to filters. In an earlier field trial, we achieved a phase drift improvement by a factor of 300 and stable classical visibility over 98% in 25 km multicore fiber in hour-long measurements[24]. It is worth mentioning that the same field trial also revealed long-term stable polarization[24]. However, since the quantum signal and the monitor signal pass together through the state encoder, the monitor signal might receive an undesired modulation. This was avoided by aligning the polarization of the monitor signal to the extra-ordinary axis of electro-optic modulators (EOMs) used in the setup, along which the electro-optic modulation coefficient is much smaller than the ordinary axis. We selected the two wavelengths $\lambda_Q = 1550.92$ nm and $\lambda_M = 1554.13$ nm for the quantum and monitor signals, respectively.

The main components of the experimental setup shown in Supplementary Fig. 4 are the transmitter, sketched in Alice's inset, and the receiver, sketched in Bob's inset pair.

At the transmitter side, a weak coherent pulse (WCP) source generates time-bin qubits at 487 MHz by carving a CW laser (Q laser) into a train of 120-ps pulses. Performing the carving on CW light with two concatenated intensity modulators (IM) provides high extinction ratio while guaranteeing zero initial relative phase difference. The pulsed laser signal and the monitor signal (M laser in the figure) are multiplexed in a 2×2 beam-splitter (BS), and further modulated prior to transmission in the fiber cores. To this end, two additional IM modulators and a phase modulator driven by RF signals generated by a field-programmable gate array (FPGA) are used. In particular, the $\mathcal{Z}$ states are generated by letting the signals through both cores and by applying the phase $\phi = \{0, \pi\}$ to the signal injected in core A. On the other hand, the $\mathcal{X}$ states are generated by blocking the signal at the input of either one of the two cores, and by applying a relative phase $\phi = \{0, \pi\}$ between two consecutive pulses at the input of core A.

Here by $|\cap -\rangle_X$ ($|- \cap\rangle_X$) we denote a state consisting of a pulse in the first (second) time bin, injected in the fiber core X (where $X = A, B$). The state $|\cap^\phi \cap\rangle_X$ consists of two pulses with a phase difference ϕ, each occupying one bin of the time-bin state. The two arms are balanced in power and further, a variable optical attenuator (VOA) sets the mean photon number per qudit state to single photon level at an optimal value derived from optimization considering security parameters, channel, and receiver's specifications. A detailed description of the setup is given in the section Method.

For the channel, a bundle of four deployed multicore fibers[21], each with four nominally uncoupled cores and a length of ≈ 6.5 km are concatenated to form a 26-km-long fiber link. In addition, we looped back two cores, forming an equivalent 52-km 2-core fiber link. This is sketched in Supplementary Fig. 4, while the characteristics of the deployed multicore fiber

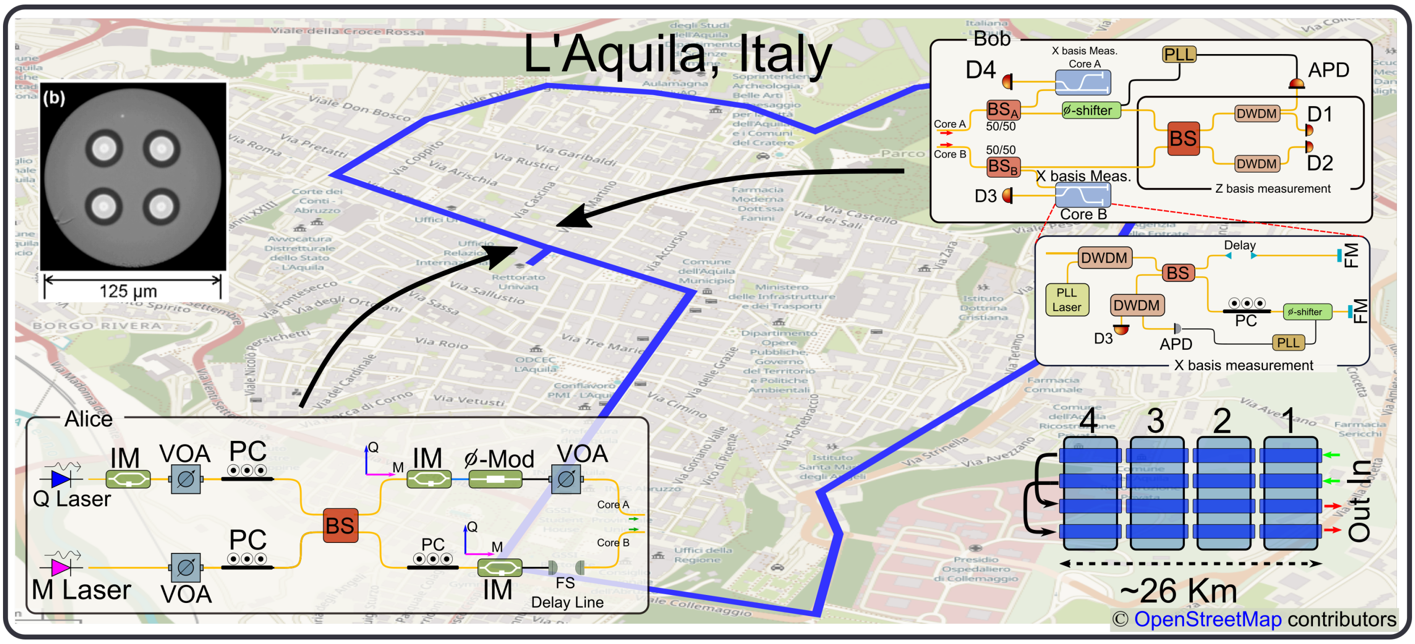

Supplementary Figure 4: **Illustration of the setup.** Schematic of transmitter (Alice), receiver (Bob) both located at the University of L'Aquila headquarters, and multicore fiber channel. **Transmitter.** Q (M) Laser: Quantum (Monitor) Laser, IM: Intensity modulator, VOA: Variable Optical Attenuator, PC: Polarization Controller, ϕ-Mod: Phase Modulator, BS: Beam-splitter, FS: Free-Space. **Receiver.** PLL: Phase-Locked Loop, ϕ-shifter: Piezo Phase Shifter, DWDM: Dense Wavelength Division Multiplexer, APD: Avalanche Photo-Diode, FM: Faraday Mirror. **Channel.** Channel is formed by concatenating four multicore fibers with four uncoupled cores, each of approximately 6.5-km length. At the endpoint, two cores are connected back into the others, resulting in a 52-km-long two-core fiber link. The multicore fibers are deployed in an underground tunnel in the historical downtown area of the city of L'Aquila, Italy. The map depicting the city of L'Aquila and the span of the multicore fiber is roughly drawn based on the exact map presented in[21]. **b)** The cross-section of the MCF. The four cores and the trench design are visible in the photo[21].

are given in the section Method.

At the receiver side, Bob passively selects the basis of measurement and extracts the quantum signals with a 50-GHz grid dense wavelength-division multiplexer (DWDM) of the kind used for dense wavelength division multiplexed fiber-optic communication systems. To read the encoded information, interference of the constituent pulses of transmitted qudit states is performed. For the $\mathcal{Z}$ basis, the two cores, stabilized with the PLL, interfere in a 50:50 beam-splitter. The outputs are filtered prior to detection and the monitor signal is fed back to the phase-locked loop for stabilization. The quantum signal is detected by detectors D1 and D2. The $\mathcal{X}$-basis measurement is carried out with an unbalanced Michelson interferometer stabilized with the same technique, and the outcome is detected with detectors D3 and D4. All detectors are superconducting nanowire single-photon detectors operating at 2 K, with 92% detection efficiency at 330 Hz dark count and 25 ps jitter. A time-to-digital (TDC) converter with 1 ps resolution registered the detection events for post-processing and QBER analysis.

Note that the transmitter setup used in this work can generate all states but the $\mathcal{X}$ state $|--\rangle_A + |\cap^\pi \cap\rangle_B$, where state normalization is omitted to simplify the notation, here as well as throughout the rest of the paper. However, since the $\mathcal{X}$-basis is only used for security check, this is not a limitation with respect to the goal of demonstrating the proposed scheme[25]. On the other hand, the receiver can receive all states, with the caveat that the phase modulator in the $\mathcal{X}$-basis branch must impose a phase equal to zero or $\pi/2$ per pass, depending on which type of $\mathcal{X}$ state is to be received. This is because the backward-propagating signal in the upper arm of the beam-splitter is lost in the absence of an optical circulator.

[1] C. C. W. Lim, M. Curty, N. Walenta, F. Xu, and H. Zbinden, Phys. Rev. A **89**, 022307 (2014).

[2] I. Vagniluca, B. Da Lio, D. Rusca, D. Cozzolino, Y. Ding, H. Zbinden, A. Zavatta, L. K. Oxenløwe, and D. Bacco, Phys. Rev. Appl. **14**, 014051 (2020).

[3] R. Mueller, D. Ribezzo, M. Zahidy, L. K. Oxenløwe, D. Bacco, and S. Forchhammer, "Efficient information reconciliation for high-dimensional quantum key distribution," (2023), arXiv:2307.02225 [quant-ph].

[4] H.-K. Mao, Q. Li, P.-L. Hao, B. Abd-El-Atty, and A. M. Iliyasu, Opt Quant Electron **54**, 163 (2022).

[5] G. Cañas, N. Vera, J. Cariñe, P. González, J. Cardenas, P. W. R. Connolly, A. Przysiezna, E. S. Gómez, M. Figueroa, G. Vallone, P. Villoresi, T. F. da Silva, G. B. Xavier, and G. Lima, Phys. Rev. A **96**, 022317 (2017).

[6] S. Etcheverry, G. Cañas, E. S. Gómez, W. A. T. Nogueira, C. Saavedra, G. B. Xavier, and G. Lima, Sci Rep **3**, 2316 (2013).

[7] M. Mirhosseini, O. S. Magaña-Loaiza, M. N. O'Sullivan, B. Rodenburg, M. Malik, M. P. J. Lavery, M. J. Padgett, D. J. Gauthier, and R. W. Boyd, New J. Phys. **17**, 033033 (2015).

[8] J. Liu, Z. Lin, D. Liu, X. Feng, F. Liu, K. Cui, Y. Huang, and W. Zhang, "High-dimensional quantum key distribution using energy-time entanglement over 242 km partially deployed fiber," (2022), arXiv:2212.02695 [quant-ph].

[9] T. Zhong, H. Zhou, R. D. Horansky, C. Lee, V. B. Verma, A. E. Lita, A. Restelli, J. C. Bienfang, R. P. Mirin, T. Gerrits, S. W. Nam, F. Marsili, M. D. Shaw, Z. Zhang, L. Wang, D. Englund, G. W. Wornell, J. H. Shapiro, and F. N. C. Wong, New J. Phys. **17**, 022002 (2015).

[10] C. Lee, D. Bunandar, Z. Zhang, G. R. Steinbrecher, P. B. Dixon, F. N. C. Wong, J. H. Shapiro, S. A. Hamilton, and D. Englund, Opt. Express **27**, 17539 (2019).

[11] N. T. Islam, C. C. W. Lim, C. Cahall, J. Kim, and D. J. Gauthier, Sci. Adv. **3**, e1701491 (2017).

[12] Y. Jo, H. S. Park, S.-W. Lee, and W. Son, Entropy **21** (2019), 10.3390/e21010080.

[13] I. Ali-Khan, C. J. Broadbent, and J. C. Howell, Phys. Rev. Lett. **98**, 060503 (2007).

[14] M. Mafu, A. Dudley, S. Goyal, D. Giovannini, M. McLaren, M. J. Padgett, T. Konrad, F. Petruccione, N. Lütkenhaus, and A. Forbes, Phys. Rev. A **88**, 032305 (2013).

[15] Y. Ding, D. Bacco, K. Dalgaard, X. Cai, X. Zhou, K. Rottwitt, and L. K. Oxenløwe, npj Quantum Inf **3**, 25 (2017).

[16] D. Bacco, B. D. Lio, D. Cozzolino, Y. Ding, M. Galili, K. Rottwitt, and L. K. Oxenløwe, in *Quantum and Nonlinear Optics VI*, Vol. 11195, edited by Q. Gong, G.-C. Guo, and B. S. Ham, International Society for Optics and Photonics (SPIE, 2019) p. 111950G.

[17] W. Li, L. Zhang, H. Tan, Y. Lu, S.-K. Liao, J. Huang, H. Li, Z. Wang, H.-K. Mao, B. Yan, Q. Li, Y. Liu, Q. Zhang, C.-Z. Peng, L. You, F. Xu, and J.-W. Pan, Nat. Photon. **17**, 416 (2023).

[18] F. Grünenfelder, A. Boaron, G. V. Resta, M. Perrenoud, D. Rusca, C. Barreiro, R. Houlmann, R. Sax, L. Stasi, S. El-Khoury, E. Hänggi, N. Bosshard, F. Bussières, and H. Zbinden, Nat. Photon. **17**, 422 (2023).

[19] B. Da Lio, D. Bacco, D. Cozzolino, N. Biagi, T. N. Arge, E. Larsen, K. Rottwitt, Y. Ding, A. Zavatta, and L. K. Oxenløwe, IEEE Journal of Selected Topics in Quantum Electronics **26**, 1 (2020).

[20] A. Mecozzi, C. Antonelli, M. Mazur, N. Fontaine, H. Chen, L. Dallachiesa, and R. Ryf, in *European Conference on Optical Communication (ECOC) 2022* (Optica Publishing Group, 2022) p. Tu3D.1.

[21] T. Hayashi, T. Nagashima, T. Nakanishi, T. Morishima, R. Kawawada, A. Mecozzi, and C. Antonelli, "Field-deployed multi-core fiber testbed," (2019).

[22] M. Pittaluga, M. Minder, M. Lucamarini, M. Sanzaro, R. I. Woodward, M.-J. Li, Z. Yuan, and A. J. Shields, Nat. Photon. **15**, 530 (2021).

[23] C. Agnesi, M. Avesani, L. Calderaro, A. Stanco, G. Foletto, M. Zahidy, A. Scriminich, F. Vedovato, G. Vallone, and P. Villoresi, Optica **7**, 284 (2020).

[24] D. Bacco, N. Biagi, I. Vagniluca, T. Hayashi, A. Mecozzi, C. Antonelli, L. K. Oxenløwe, and A. Zavatta, Photon. Res. **9**, 1992 (2021).

[25] N. T. Islam, C. C. W. Lim, C. Cahall, J. Kim, and D. J. Gauthier, Phys. Rev. A **97**, 042347 (2018).
